# *Arthrobacter* *siccitolerans* sp. nov., a highly desiccation-tolerant, xeroprotectant-producing strain isolated from dry soil

**DOI:** 10.1099/ijs.0.052902-0

**Published:** 2013-11

**Authors:** L. SantaCruz-Calvo, J. González-López, M. Manzanera

**Affiliations:** Institute for Water Research, and Department of Microbiology, University of Granada, Granada, Spain

## Abstract

A novel desiccation-tolerant, xeroprotectant-producing bacterium, designated strain 4J27^T^, was isolated from a *Nerium oleander* rhizosphere subjected to seasonal drought in Granada, Spain. Phylogenetic analysis based on 16S rRNA gene sequencing placed the isolate within the genus *Arthrobacter*, its closest relative being *Arthrobacter phenanthrenivorans* Shep3 DSM 18606^T^, with which it showed 99.23 % 16S rRNA gene sequence similarity. DNA–DNA hybridization measurements showed less than 25 % relatedness between strain 4J27^T^ and *Arthrobacter phenanthrenivorans* DSM 18606^T^. The DNA base composition of strain 4J27^T^ was 65.3 mol%. The main fatty acids were anteiso C_15 : 0_, anteiso C_17 : 0_, C_16 : 0_ and iso C_16 : 0_ and the major menaquinone was MK-9 (H_2_). The peptidoglycan type was A3α with an l-Lys–l-Ser–l-Thr–l-Ala interpeptide bridge. The bacterium tested positive for catalase activity and negative for oxidase activity. Phylogenetic, chemotaxonomic and phenotypic analyses indicated that the desiccation-tolerant strain 4J27^T^ represents a novel species within the genus *Arthrobacter*, for which the name *Arthrobacter* *siccitolerans* is proposed. The type strain is 4J27^T^ ( = CECT 8257^T^ = LMG 27359^T^).

The genus *Arthrobacter*, first defined by Conn & Dimmick (1947), belongs to the class *Actinobacteria* and includes Gram-stain-positive coryneform bacteria with aerobic metabolism and little or no acid production from glucose. Species of the genus *Arthrobacter* contain lysine in the peptidoglycan and have a DNA G+C content ranging from 59 mol% to 66 mol% (Keddie *et al.*, 1986; Jones & Keddie, 1992). These bacteria typically take the shape of rods in younger cultures and cocci in older cultures (Keddie *et al.*, 1986), depending on their growth rate and nutritional conditions (Germida & Cassida, 1980). The transition to this coccoid-like state has been shown to require manganese (Germida & Cassida, 1980). The small coccoid-like state has been described as being the most stable form. Due to their pleomorphic and heterogeneous appearance, strains of species of the genus *Arthrobacter* were originally grouped with the Corynebacteria (Keddie *et al.*, 1986).

In response to changing extracellular osmolarity such as desiccation or increased salinity some micro-organisms accumulate small organic compounds (Brown, 1976; Arakawa& Timasheff, 1982). These compatible solutes act as protectants, which under laboratory conditions can also stabilize enzymes, DNA, cell membranes and even whole cells against different kinds of stress, such as freezing, drying and heating (Brown, 1976; Yancey *et al.*, 1982; Knapp *et al.*, 1999; Manzanera *et al.* 2002, Narváez-Reinaldo *et al.*, 2010, Julca *et al.*, 2012). Our group has previously reported a new method for the isolation of desiccation-tolerant micro-organisms from dry soil using organic solvents as selective agents (Manzanera *et al.*, 2004a; Narváez-Reinaldo *et al.*, 2010). Strain 4J27^T^ displayed remarkably high tolerance to desiccation and produced excellent xeroprotectants for the dry stabilization of proteins (lipase enzymes) and whole prokaryotic cells (*Escherichia coli* MC4100) compared with those when trehalose was used (Manzanera *et al.*, 2004b; Narváez-Reinaldo *et al.*, 2010). Among the 10 different xeroprotectants tested, the best results were observed with S4J27-D (composed of trehalose, glutamine and glucose), a synthetic mixture derived from strain 4J27^T^ (Narváez-Reinaldo *et al.*, 2010).

Here we describe the morphological, biochemical and phylogenetic characteristics of this desiccation-tolerant strain (4J27^T^), isolated from dry soil and with a remarkable potential for the dry stabilization of some biomaterials. On the basis of the phylogenetic analysis of the 16S rRNA gene sequence together with physiological, chemotaxonomic and DNA–DNA hybridization analyses we demonstrate that strain 4J27^T^ represents a novel species of the genus *Arthrobacter*.

Strain 4J27^T^ was grown at 30 °C (±3 °C) in tryptone soya agar (TSA) plates and in tryptone soya broth (TSB) or M9 minimal medium (M6030; Sigma). *Arthrobacter phenanthrenivorans* DSM 18606^T^ was included in the study as reference.

Strain 4J27^T^, the object of this study, had already been assigned to the genus *Arthrobacter* by partial analysis of its 16S rRNA gene sequence (GenBank accession number GU815139; Narváez-Reinaldo *et al.*, 2010), which was compared with those in the EzTaxon-e server (http://eztaxon-e.ezbiocloud.net/, Kim *et al.*, 2012). The nearly complete sequence of the 16S rRNA gene of strain 4J27^T^ (approximately 1500 bp) was aligned with the sequences of closely related species of the genus *Arthrobacter* by using the clustal x 2 program (Larkin *et al.*, 2007). A phylogenetic tree was inferred using the neighbour-joining (Saitou & Nei, 1987) and maximum-likelihood (Guindon & Gascuel, 2003) methods with the mega 5.0 software package (Tamura *et al.*, 2011). Bootstrap analysis was based on 1000 resamplings (Felsenstein, 1985). The distances were calculated according to Kimura’s two-parameter model (Kimura, 1980). The resulting neighbour-joining tree obtained with Kimura’s two-parameter model is shown in Fig. 1 and the maximum-likelihood tree is shown in Fig. S1, available in IJSEM Online.

**Fig. 1.**
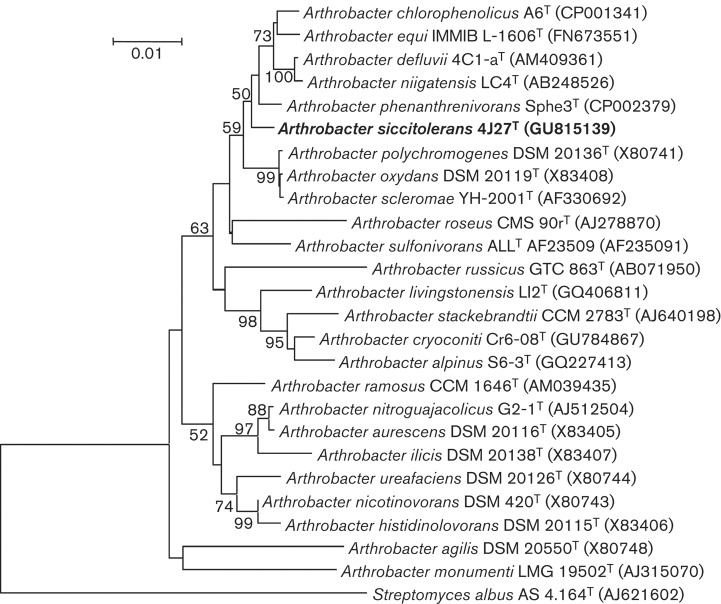
Neighbour-joining phylogenetic tree based on 16S rRNA sequence comparisons of strain 4J27^T^ and its 24 closest relatives. *Streptomyces albus* AS 4.164^T^ was used as the outgroup. The numbers at bifurcations indicate how many times each species coincided in this position as percentages and only values > 50% are shown. Bar, 0.01 changes per nucleotide position.

The sequence corresponding to the 16S rRNA gene of strain 4J27^T^ showed 99.23 % similarity to that of *Arthrobacter phenanthrenivorans* DSM 18606^T^. Phylogenetic analysis showed that strain 4J27^T^ clearly belongs to the genus *Arthrobacter**,* and its closest relative was *Arthrobacter phenanthrenivorans* DSM 18606^T^.

DNA–DNA hybridization was carried out at the Deutsche Sammlung von Mikroorganismen und Zellkulturen (DSMZ; Braunschweig, Germany). Cells of *Artrhobacter phenanthrenivorans* DSM 18606^T^ and strain 4J27^T^ were disrupted by using a French pressure cell (Thermo Spectronic) and the DNA of each strain in the crude lysate were purified by chromatography on hydroxyapatite as described by Cashion *et al.* (1977). DNA–DNA hybridization was conducted as described by De Ley *et al.* (1970) with the modifications described by Huss *et al.* (1983) using a model Cary 100 Bio UV/VIS-spectrophotometer equipped with a Peltier-thermostat-regulated 6×6 multicell charger and a temperature controller with *in situ* temperature probe (Varian). DNA–DNA hybridization of strain 4J27^T^ with *Arthrobacter phenanthrenivorans* DSM 18606^T^ resulted in a DNA–DNA relatedness value of 22.3 % (22.1 %), the value in parentheses being the result of measurements in duplicate. On the basis of DNA–DNA reciprocal hybridization, strain 4J27^T^ did not belong to the species *Arthrobacter phenanthrenivorans* according to the recommendations of a threshold value of 70 % DNA–DNA relatedness for the definition of bacterial species (Wayne *et al.*, 1987). Therefore strain 4J27^T^ probably represents a novel species of the genus *Arthrobacter*.

The G+C (mol%) content of the genomic DNA of strain 4J27^T^ was analysed at the DSMZ. The dG and dT ratio was calculated according to the method of Mesbah *et al.* (1989). Species of the genus *Arthrobacter* have previously been described as Gram-stain-positive actinobacteria with high GC content (Keddie *et al.*, 1986; Jones & Keddie ,1992), which typically have a DNA G+C content in the range of 59–66 mol% (Keddie *et al.*, 1986). The DNA G+C content of strain 4J27^T^ was 65.3 mol%, which was within the range shown by all members of the genus *Arthrobacter* and considered to have a high GC content (Keddie *et al.*, 1986).

Chemotaxonomic analyses were carried out by the Identification Service of the DSMZ. Peptidoglycans were isolated from strain 4J27^T^ and their structures analysed (Schleifer & Kandler, 1972). After derivatization according to the method of MacKenzie (1987) the approximate molar amino-acid ratio was determined by gas chromatography. Free amino groups within the peptidoglycan were detected by labelling with 1-fluoro-2,4-dinitrobenzene (Schleifer, 1985). The peptidoglycan of strain 4J27^T^ was composed of Ala, Ser, Thr, Glu and Lys at a molar ratio of 2.8 : 1.2 : 1.0 : 1.0 : 1.5. Two-dimensional TLC of the partial hydrolysate (4 M HCl, 100 °C, 45 min) of the peptidoglycan revealed the presence of the peptides l-Ala–d-Glu, l-Lys–d-Ala, l-Lys–l-Ser, l-Lys–l-Ser–l-Thr, d-Ala–l-Lys–l-Ser–l-Thr, l-Ser–l-Thr and l-Ala–d-Ala. On the basis of these results it was concluded that strain 4J27^T^ contains a type A3α peptidoglycan (Schleifer & Kandler, 1972) with an l-Lys–l-Ser–l-Thr–l-Ala interpeptide bridge (A11.23 DSMZ-Catalogue of strains, 7th edition, 2001), which is found in the more closely related members of the genus *Arthrobacter*, according to the neighbour-joining phylogenetic tree, such as *Arthrobacter chlorophenolicus*, *Arthrobacter oxydans*, *Arthrobacter polychromogenes*, *Arthrobacter sulfonivorans*, *Arthrobacter equi*, *Arthrobacter niigatensis*, *Arthrobacter phenanthrenivorans*, *Arthrobacter defluvii*, *Arthrobacter roseus* and *Arthrobacter scleromae* (Borodina *et al.*, 2002; Kodama *et al.*, 1992; Westerberg *et al.*, 2000; Reddy *et al.*, 2002; Huang *et al.*, 2005; Kim *et al.*, 2008; Ding *et al.*, 2009; Yassin *et al.*, 2011). Strains containing a type A3α peptidoglycan make up a rather uniform group, although they do show a considerable number of different types of interpeptide bridge. Most of these strains belong to the genus *Arthrobacter* and are distinguished by strictly aerobic growth and a complete life cycle (Conn & Dimmick, 1947; Schleifer & Kandler, 1972).

Fatty-acid methyl esters were obtained from 40 mg cells of strain 4J27^T^ scraped from Petri dishes by saponification, methylation and extraction using the methods of Miller (1982) and Kuykendall *et al.*, (1988) with minor modifications. The fatty-acid methyl-ester mixtures were separated using the Sherlock Microbial Identification System (MIS) (MIDI, Microbial ID). The main cellular fatty acids of the highly desiccation-tolerant strain 4J27^T^ were, from highest to lowest, anteiso-C_15 : 0_, 41.20 %; anteiso-C_17 : 0_, 30.86 %; C_16 : 0_, 10.21 %; iso-C_16 : 0_, 6.61 %; iso-C_15 : 0_, 4.40 %; C_18 : 0_, 2.38 %; iso-C_17 : 0_, 1.79 %; iso-C_14 : 0_, 0.83 %; C_14 : 0_, 0.75 %; anteiso-C_19 : 0_, 0.61 % and iso-C_18 : 0_, 0.36 %. The fatty-acid composition of strain 4J27^T^ was consistent with that of the genus *Arthrobacter*, with branched-chain fatty acid, antesio-pentadecanoic acid (anteiso-C_15 : 0_) predominating (Westerberg *et al.*, 2000).

Respiratory quinones were analysed as described by Tindall (1990a; b), using TLC and UV mass spectroscopy, and found menaquinone to be the sole quinone component. Analyses of the electron-transport system (isoprenoid quinones) for strain 4J27^T^ resulted in detection of MK9 (II-H_2_) 68 %; MK9 21 % and MK8 (II-H_2_) 11 %.

To analyse the whole cell sugars of strain 4J27^T^, cells were hydrolysed in 0.5 M H_2_SO_4_ for 2 h at 100 °C. Sulfuric acid was removed by 20 % *N,N*-dioctylmethylamine in chloroform according to the method of Whiton *et al.* (1985). Sugars in the hydrolysate were analysed by TLC on cellulose plates according to the methods of Staneck & Roberts (1974). The whole-cell sugars of the isolated strain were galactose, glucose, mannose, ribose and rhamnose.

Mobility was tested by stab-inoculating mannitol-mobility semi-solid agar (413782; Ultimed). This semi-solid agar medium enabled us to analyse the nitrate reductase activity (capacity to reduce nitrate to nitrite) and catabolism of mannitol by using Griess–Ilosvay A and B reagents. Oxidase activity was determined using 1 % w/v *N*,*N*,*N*′,*N*′-tetramethyl-*p*-phenylenediamine and catalase activity was determined by the production of bubbles from 3 % v/v. H_2_O_2_. Cells of strain 4J27^T^ were identified as catalase-positive, oxidase-negative, nitrate reductase-negative and mannitol-positive. *Arthrobacter phenanthrenivorans* DSM 18606^T^.

To characterize the growth of strain 4J27^T^ at different temperatures, pH values and salinities, cultures were incubated at 150 r.p.m. in Luria–Bertani (LB) rich medium (L3152; Sigma). Cell growth was monitored at different temperatures (5, 10, 15, 20, 25, 30, 35, 40, 45 and 50 °C), pH (3, 5, 7, 9, 12 and 13) and NaCl concentrations (0, 0.2, 0.4, 0.6, 0.8, 1 and 1.2 M) by measuring the OD_600_ in triplicate at 0, 12 and 24 h using a UV-160A spectrophotometer (Shimadzu). Strain 4J27^T^ grew best at 30 °C in LB medium. It was able to grow at 37 °C and 15 °C but not at 40 °C or 10 °C. The pH range for growth was between 5 and 9 with optimum growth at pH 7. Strain 4J27^T^ grew in NaCl concentrations ranging from 0 to 0.8 M but grew best at 0.2 M. This differed clearly from the most closely related species, *Arthrobacter phenanthrenivorans* DSM 18606^T^, which was able to grow at 4 °C but not at pH 5.

The following API kits were used for testing, API Coryne, API 20NE and API 20E (bioMérieux,). Each test was interpreted according to the manufacturer’s instructions. Biolog tests were performed to investigate which compounds the strains in question could use for respiration. A GP2 MicroPlate (Cat. No 1014; Biolog), containing 95 different carbon compounds, was used to test for substrate oxidation. The chemistry of these plates is based on tetrazolium reduction, in response to metabolic processes such as fermentation and oxidation. Tetrazolium reduction produced formazan in a variety of colours from dark blue to deep red to orange, depending upon the original tetrazolium salt used as the substrate for the reaction. MicroPlates were inoculated and interpreted according to the manufacturer’s instructions. The results were recorded after 12 h based on *A*_585_. Antibiotic susceptibility testing was performed using the disc-diffusion method in which the antibiotic diffuses away from the disc in two dimensions, forming a concentration gradient that inhibits the growth of bacteria and causes an inhibition zone (Piddock, 1990). The results were interpreted according to the criteria established for staphylococci in 1997 by the National Committee for Clinical Laboratory Standards (2000). At the concentrations assayed, the inhibition zone caused by streptomycin was 157 mm, rifampicin 347 mm, chloramphenicol 340 mm, kanamycin 150 mm and tetracycline 157 mm and thus it could be concluded that strain 4J27^T^ was susceptible to all the antibiotics tested. The phenotypic differences between strain 4J27^T^ and closely related species are summarized in Table 1 and the physiological differences between strain 4J27^T^ and its closest relative species *Arthrobacter phenanthrenivorans* DSM 18606^T^ are summarized in Table S1.

**Table 1.** Differential characteristics between strain 4J27^T^ and the type strains of the most closely related species of the genus *Arthrobacter* Strains: 1, 4J27^T^; 2, *Arthrobacter phenanthrenivorans* DSM 18606^T^; 3, *Arthrobacter niigatensis* IAM 15382^T^; 4, *Arthrobacter. defluvii* DSM 18782^T^; 5. *Arthrobacter equi* DSM 23395^T^; 6. *Arthrobacter chlorophenolicus* DSM 12829^T^; 7. *Arthrobacter polychromogenes* DSM 20136^T^; 8. *Arthrobacter oxydans* DSM 20119^T^; 9. *Arthrobacter scleromae* JCM 12642^T^. Data of the reference species were taken from Kallimanis *et al.* (2009), Ding *et al.* (2009), Kim *et al.*, (2008), Yassin *et al.* (2011), Westerberg *et al.* (2000), Schippers-Lammertse *et al.* (2009), Sguros (1955), Huang *et al.* (2005) and the present study. +, Positive; −, negative; nd, not determined; CFA, cellular fatty acid.

Trait	1	2	3	4	5	6	7	8	9
Motility	−	−	−	−	−	+	−	−	−
Colony colour	Cream	Yellowish	Grey/yellow	Creamy white	Cream	Grey	Blue-green	Grey-white	White
Temperature	15–35	4–37	5–40	5–37	10–35	3–37	10–37	20–35	15–37
pH	5–9	6.5–8.5	6–11	6–10	6–9	5–9	6–11	5–9	6–9
Reduction of nitrate	+	+	+	+	−	−	+	+	+
Hydrolysis of gelatin	−	−	+	−	+	+	+	+	+
Utilization of:									
Maltose	+	−	−	−	+	+	+	+	+
d-Ribose	+	−	+	+	−	+	+	+	−
Sucrose	+	+	+	−	+	+	+*	+	+
Trehalose	+	−*	+	−	+	+*	−*	−*	+
d-Xylose	+	+	−	−	+	−	+	+	+
d-Alanine	−	+*	nd	−	+	+	+*	−*	nd
Glucose-1-phosphate	+	−*	+	nd	nd	−*	−*	−*	nd
Inulin	+	−*	nd	−	nd	−	−*	−	+
Major CFAs	antesio-C_15 : 0_ antesio-C_17 : 0_	antesio-C_15 : 0_ iso-C_15 : 0_	antesio-C_15 : 0_ antesio-C_17 : 0_	anteiso-C_15 : 0_ iso-C_16 : 0_	anteiso-C_15 : 0_ iso-C_15 : 0_	anteiso-C_15 : 0_ iso-C_16 : 0_	anteiso-C_15 : 0_ antesio-C_17 : 0_	anteiso-C_15 : 0_ anteiso-C_15 : 0_	anteiso-C_15 : 0_ iso-C_15 : 0_
Major menaquinone	MK-9 (H_2_)	MK-8 (H_2_)	MK-9 (H_2_)	MK-9 (H_2_)	MK-9 (H_2_)	MK-9 (H_2_)	MK-9 (H_2_)	MK-9 (H_2_)	MK-8 (H_2_)
DNA G+C content (mol%)	65.3	67.5	70.8	63.5–64.4	67.0	65**±**1	62.9	63.1	64.7

* Data taken from the present study.

The degree of tolerance to desiccation shown by strain 4J27^T^ was compared with that of the previously described desiccation-tolerant bacteria *Acinetobacter calcoaceticus* PADD68 (Narváez-Reinaldo *et al.*, 2010), the desiccation-sensitive strain *Pseudomonas putida* KT2440 (Manzanera *et al.*, 2002) and the closely related *Arthrobacter phenanthrenivorans* DSM 18606^T^. A colony of a pure culture grown for 48 h of each strain, containing 10^7^ to 10^9^ cells, was suspended in 1 ml M9 minimal medium. Aliquots (100 µl) were placed on sterile Petri dishes and dried under a current of sterile air for 24 h. The cells were then suspended in 1 ml sterile saline buffer, and serial dilutions of the cell suspension were plated on TSA plates before and after drying. All such procedures were conducted at room temperature. The survival rate was calculated in terms of c.f.u. ml^−1^ after drying compared with c.f.u. ml^−1^ before drying, expressed as a percentage. The assays were performed in triplicate accordingly to the protocol of Manzanera *et al.*, 2002. Strain 4J27^T^ showed the highest values of desiccation tolerance (31.58 %±6.9 %), which were significantly different from those of the positive control, *Acinetobacter calcoaceticus* PADD68 (3.23 %±0.2 %) and more so from the closely related strain, *Arthrobacter phenanthrenivorans* DSM 18606^T^ (1.5 %±0.41 %). As expected, the desiccation tolerance of the negative control, *P. putida* KT2440^T^, was below detectable levels. Therefore the closely related species *Arthrobacter phenanthrenivorans* DSM 18606^T^ is considered to be desiccation-sensitive, due to its low degree of desiccation tolerance, in contrast to the novel strain, which is considered to be a desiccation-tolerant strain.

On the basis of phylogenetic analysis of its 16S rRNA gene sequence, together with physiological, chemotaxonomic and DNA–DNA hybridization analyses, strain 4J27^T^ is considered to represent a novel species of the genus *Arthrobacter*, for which the name *Arthrobacter* *siccitolerans* is proposed.

## Description of *Arthrobacter* *siccitolerans* sp. nov.

*Arthrobacter* *siccitolerans* (sic.ci.to′le.rans. L. adj. *siccus* dry, L. part. adj. *tolerans* tolerating; N.L. part. adj. *siccitolerans* dry-tolerating).

Cells are non-motile, non-spore-forming, Gram-positive, aerobic and rod-to-coccus-shaped. Colonies on TSA are convex, circular, cream, opaque and usually 1–2 mm in diameter within 2 days at 30 °C. Catalase-positive, oxidase-negative and nitrate-reductase-negative (no capacity to reduce nitrate to nitrite). Grows at temperatures from 15 to 35 °C, pH 5–9 and with 0–0.8 M NaCl in LB medium. The peptidoglycan type is A3α (Schleifer & Kandler, 1972), with an l-Lys–l-Ser–l-Thr–l-Ala interpeptide bridge. The major cellular fatty acids are anteiso C_15 : 0_, anteiso C_17 : 0_, C_16 : 0_ and iso C_16 : 0_. The major menaquinone is MK9-(II-H_2_). The whole-cell sugars of the strain are galactose, glucose, mannose, ribose and rhamnose. It reduces nitrites to nitrogen. Indole and acetoin (Voges–Proskauer) production are positive. According to the results from the API CORYNE, API 20NE and API 20E strips, the following enzyme activities are detected: pirazinamidase, β-glucuronidase, β-galactosidase, α-glucosidase, β-glucosidase (aesculin), β-galactosidase (*p*-nitrophenyl-β-d-galactopyranosidase). Assimilation of glucose, arabinose, mannose, mannitol, N-acetyl-glucosamine, maltose, potassium gluconate, malate, trisodium citrate, inositol, sorbitol, rhamnose, sucrose, melibiose, amygdalin and arabinose are positive. The following enzyme activities are not present: β-galactosidase (*o*-nitro-phenyl-β-d-galactopyranoside), arginine dihydrolase, lysine decarboxylase, ornithine decarboxylase, urease, tryptophan desaminase, gelatinase, pyrrolidonyl arylamidase, alkaline phosphatase and *N*-acetyl-β-glucosaminidase. Production of H_2_S is negative and does not use citrate. In the Biolog GP2 MicroPlates the following substrates were used for respiration: dextrin, inulin, l-arabinose, *N*-acetyl-d-glucosamine, *N*-acetyl-d-mannosamine, d-arbutin, cellobiose, d-fructose, d-galactose, d-galacturonic acid, α-d-glucose, gentiobiose, lactamide, l-lactic acid, lactulose, maltose, maltotriose, d-mannitol, d-mannose, melezitose, melibiose, 3-methyl glucose, α-methyl d-mannoside, palatinose, d-psicose, d-rafinose, l-rhamnose, d-ribose, salicin, d-sorbitol, sucrose, trehalose, turanose, xylitol, d-xylose, acetic acid, α-hydroxybutyric acid, β-hydroxybutyric acid, *p*-hydroxyphenylacetic acid, α-ketovaleric acid, l-malic acid, pyruvic acid, l-alaninamide, l-alanyl glycine, glycyl-l-glutamic acid, putrescine, glycerol, adenosine, 2′-deoxy adenosine, inosine, thymidine, uridine, thymidine-5′ monophosphate, glucose-1-phosphate and d-l-α-glycerol phosphate. The following compounds were not used for respiration: α-cyclodextrin, β-cyclodextrin, glycogen, mannan, Tween 40, Tween 80, amygdalin, d-arabitol, d-fructose, l-fucose, d-gluconic acid, *myo*-inositol, α-d-lactose, α-methyl-d-galactoside, β-methyl-d-galactoside, α-methyl-d-glucoside, β-methyl-d-glucoside, palatinose, propionic acid, l-alanine, l-asparagine, l-glutamic acid, l-pyroglutamic acid and l-serine, sedoheptulose, stachyose, d-tagatose, γ-hydroxybutyric acid, α-ketoglutaric acid, d-lactic acid methyl ester, d-malic acid, methyl pyruvate, mono-methyl succinate, succinamic acid, succinic acid, d-alanine, *N*-acetyl-l-glutamic acid, 2,3-butanediol, adenosine-5′-monophosphate, uridine-5′-monophosphate, fructose-6-phosphate and glucose-6-phosphate. Susceptible to all the antibiotics tested: streptomycin, rifampicin, chloramphenicol, kanamycin and tetracycline.

The type strain, 4J27^T^ ( = CECT 8257^T^ = LMG 27359^T^), was isolated from a *Nerium oleander* rhizosphere subjected to seasonal drought in Granada, Spain. The DNA G+C content of strain 4J27^T^ is 65.3 mol%.

## References

[r2] Arakawa T. Timasheff S. N. **(**1982**).** Stabilization of protein structure by sugars. Biochemistry 21, 6536–6544 10.1021/bi00268a033 7150574

[r3] Borodina E. Kelly D. P. Schumann P. Rainey F. A. Ward-Rainey N. L. Wood A. P. **(**2002**).** Enzymes of dimethylsulfone metabolism and the phylogenetic characterization of the facultative methylotrophs *Arthrobacter sulfonivorans* sp. nov., *Arthrobacter methylotrophus* sp. nov., and *Hyphomicrobium sulfonivorans* sp. nov. Arch Microbiol 177, 173–183 10.1007/s00203-001-0373-3 11807567

[r4] Brown A. D. **(**1976**).** Microbial water stress. Bacteriol Rev 40, 803–846 1008746 10.1128/br.40.4.803-846.1976 PMC413986

[r5] Cashion P. Holder-Franklin M. A. McCully J. Franklin M. **(**1977**).** A rapid method for the base ratio determination of bacterial DNA. Anal Biochem 81, 461–466 10.1016/0003-2697(77)90720-5 907108

[r6] Conn H. J. Dimmick I. **(**1947**).** Soil bacteria similar in morphology to *Mycobacterium* and *Corynebacterium*. J Bacteriol 54, 291–303 16561362 10.1128/jb.54.3.291-303.1947 PMC526554

[r7] De Ley J. Cattoir H. Reynaerts A. **(**1970**).** The quantitative measurement of DNA hybridization from renaturation rates. Eur J Biochem 12, 133–142 10.1111/j.1432-1033.1970.tb00830.x 4984993

[r8] Ding L. Hirose T. Yokota A. **(**2009**).** Four novel *Arthrobacter* species isolated from filtration substrate. Int J Syst Evol Microbiol 59, 856–862 10.1099/ijs.0.65301-0 19329620

[r5a] DSMZ **(**2001**).** Catalogue of Strains, 7th edn, p. 617 Braunschweig: DSMZ http://www.dsmz.de/fileadmin/Bereiche/Microbiology/Dateien/Key_to_Murein2.pdf

[r9] Felsenstein J. **(**1985**).** Confidence limits on phylogenies: an approach using the bootstrap. Evolution 39, 783–791 10.2307/2408678 28561359

[r10] Germida J. J. Casida L. E. Jr **(**1980**).** Myceloid growth of *Arthrobacter globiformis* and other *Arthrobacter* species. J Bacteriol 144, 1152–1158 6254945 10.1128/jb.144.3.1152-1158.1980 PMC294782

[r11] Guindon S. Gascuel O. **(**2003**).** A simple, fast, and accurate algorithm to estimate large phylogenies by maximum likelihood. Syst Biol 52, 696–704 10.1080/10635150390235520 14530136

[r12] Huang Y. Zhao N. He L. Wang L. Liu Z. You M. Guan F. **(**2005**).** *Arthrobacter scleromae* sp. nov. isolated from human clinical specimens. J Clin Microbiol 43, 1451–1455 10.1128/JCM.43.3.1451-1455.2005 15750131 PMC1081264

[r13] Huss V. A. R. Festl H. Schleifer K. H. **(**1983**).** Studies on the spectrophotometric determination of DNA hybridization from renaturation rates. Syst Appl Microbiol 4, 184–192 10.1016/S0723-2020(83)80048-4 23194591

[r14] Jones D. Keddie R. M. **(**1992**).** The genus *Arthrobacter*. In The Prokaryotes: a Handbook on the Biology of Bacteria: Ecophysiology, Isolation, Identification, Applications, 2nd edn, vol. 2, pp. 1283–1299 Edited by Balows A. Truper H. G. Dworkin M. Harder W. Schleifer K. H. NY: Springer

[r16] Julca I. Alaminos M. González-López J. Manzanera M. **(**2012**).** Xeroprotectants for the stabilization of biomaterials. Biotechnol Adv 30, 1641–1654 10.1016/j.biotechadv.2012.07.002 22814234

[r17] Kallimanis A. Kavakiotis K. Perisynakis A. Spröer C. Pukall R. Drainas C. Koukkou A. I. **(**2009**).** *Arthrobacter phenanthrenivorans* sp. nov., to accommodate the phenanthrene-degrading bacterium *Arthrobacter* sp. strain Sphe3. Int J Syst Evol Microbiol 59, 275–279 10.1099/ijs.0.000984-0 19196765

[r18] Keddie R. M. Collins M. D. Jones D. **(**1986**).** Genus *Arthrobacter* Conn and Dimmick 1947, 300AL. In Bergey's Manual of Systematic Bacteriology, pp. 1288–1301 Edited bySneath P. H. A. Mair N. S. Sharpe M. E. Holt J. G. Baltimore: Williams & Wilkins

[r19] Kim K. K. Lee K. C. Oh H. M. Kim M. J. Eom M. K. Lee J. S. **(**2008**).** *Arthrobacter defluvii* sp. nov., 4-chlorophenol-degrading bacteria isolated from sewage. Int J Syst Evol Microbiol 58, 1916–1921 10.1099/ijs.0.65550-0 18676480

[r20] Kim O. S. Cho Y. J. Lee K. Yoon S. H. Kim M. Na H. Park S. C. Jeon Y. S. Lee J. H. **& other authors (**2012**).** Introducing EzTaxon-e: a prokaryotic 16S rRNA gene sequence database with phylotypes that represent uncultured species. Int J Syst Evol Microbiol 62, 716–721 10.1099/ijs.0.038075-0 22140171

[r21] Kimura M. **(**1980**).** A simple method for estimating evolutionary rates of base substitutions through comparative studies of nucleotide sequences. J Mol Evol 16, 111–120 10.1007/BF01731581 7463489

[r22] Knapp S. Ladenstein R. Galinski E. A. **(**1999**).** Extrinsic protein stabilization by the naturally occurring osmolytes β-hydroxyectoine and betaine. Extremophiles 3, 191–198 10.1007/s007920050116 10484175

[r23] Kodama Y. Yamamoto H. Amano N. Amachi T. **(**1992**).** Reclassification of two strains of *Arthrobacter oxydans* and proposal of *Arthrobacter nicotinovorans* sp. nov. Int J Syst Bacteriol 42, 234–239 10.1099/00207713-42-2-234 1581183

[r24] Kuykendall L. D. Roy M. A. O'Neill J. J. Devine T. E. **(**1988**).** Fatty acids, antibiotic resistance, and deoxyribonucleic acid homology groups of *Bradorhizobium japonicum*. Int J Syst Bacteriol 38, 358–361 10.1099/00207713-38-4-358

[r25] Larkin M. A. Blackshields G. Brown N. P. Chenna R. McGettigan P. A. McWilliam H. Valentin F. Wallace I. M. Wilm A. **& other authors (**2007**).** clustal w and clustal_x version 2.0. Bioinformatics 23, 2947–2948 10.1093/bioinformatics/btm404 17846036

[r4a] MacKenzie S. L. **(**1987**).** Gas chromatographic analysis of amino acids as the N-heptafluorobutyryl isobutyl esters. J Assoc Anal Chem 70, 151–160 3558269

[r26] Manzanera M. García de Castro A. Tøndervik A. Rayner-Brandes M. Strøm A. R. Tunnacliffe A. **(**2002**).** Hydroxyectoine is superior to trehalose for anhydrobiotic engineering of *Pseudomonas putida* KT2440. Appl Environ Microbiol 68, 4328–4333 10.1128/AEM.68.9.4328-4333.2002 12200283 PMC124095

[r27] Manzanera M. Vilchez S. Tunnacliffe A. **(**2004a**).** Plastic encapsulation of stabilized *Escherichia coli* and *Pseudomonas putida*. Appl Environ Microbiol 70, 3143–3145 10.1128/AEM.70.5.3143-3145.2004 15128579 PMC404431

[r28] Manzanera M. Vilchez S. Tunnacliffe A. **(**2004b**).** High survival and stability rates of *Escherichia coli* dried in hydroxyectoine. FEMS Microbiol Lett 233, 347–352 10.1111/j.1574-6968.2004.tb09502.x 15063506

[r29] Mesbah M. Premachandran U. Whitman W. **(**1989**).** Precise measurement of the G+C content of deoxyribonucleic acid by high performance liquid chromatography. Int J Syst Bacteriol 39, 159–167 10.1099/00207713-39-2-159

[r30] Miller L. T. **(**1982**).** Single derivatization method for routine analysis of bacterial whole-cell fatty acid methyl esters including hydroxy acids. J Clin Microbiol 16, 584–586 7130373 10.1128/jcm.16.3.584-586.1982 PMC272420

[r33] Narváez-Reinaldo J. J. Barba I. González-López J. Tunnacliffe A. Manzanera M. **(**2010**).** Rapid method for isolation of desiccation-tolerant strains and xeroprotectants. Appl Environ Microbiol 76, 5254–5262 10.1128/AEM.00855-10 20562279 PMC2916496

[r34] National Committee for Clinical Laboratory Standards **(**1997**).** Minimum inhibitory concentration (MIC) interpretive standards (g/ml) for organisms other than *Haemophilus* spp., *Neisseria gonorrhoeae*, and *Streptococcus* spp. NCCLS document M7–A4. Wayne, PA: National Committee for Clinical Laboratory Standards

[r35] Piddock L. J. V. **(**1990**).** Techniques used for the determination of antimicrobial resistance and sensitivity in bacteria. J Appl Bacteriol 68, 307–318 10.1111/j.1365-2672.1990.tb02880.x 2190965

[r36] Reddy G. S. N. Prakash J. S. S. Matsumoto G. I. Stackebrandt E. Shivaji S. **(**2002**).** *Arthrobacter roseus* sp. nov., a psychrophilic bacterium isolated from an Antarctic cyanobacterial mat sample. Int J Syst Evol Microbiol 52, 1017–1021 10.1099/ijs.0.02131-0 12054218

[r37] Saitou N. Nei M. **(**1987**).** The neighbor-joining method: a new method for reconstructing phylogenetic trees. Mol Biol Evol 4, 406–425 3447015 10.1093/oxfordjournals.molbev.a040454

[r38] Schippers-Lammertse A. F. Muijsers A. O. Klatser-Oedekerk K. B. **(**1963**).** *Arthrobacter polychromogenes* nov. spec., its pigments, and a bacteriophage of this species. Antonie van Leeuwenhoek 29, 1–15 10.1007/BF02046033 14024588

[r39] Schleifer K. H. **(**1985**).** Analysis of the chemical composition and primary structure of murein. Methods Microbiol 18, 123–156 10.1016/S0580-9517(08)70474-4

[r40] Schleifer K. H. Kandler O. **(**1972**).** Peptidoglycan types of bacterial cell walls and their taxonomic implications. Bacteriol Rev 36, 407–477 4568761 10.1128/br.36.4.407-477.1972 PMC408328

[r41] Sguros P. L. **(**1955**).** Microbial transformations of the tobacco alkaloids. I. Cultural and morphological characteristics of a nicotinophile. J Bacteriol 69, 28–37 13233163 10.1128/jb.69.1.28-37.1955 PMC357463

[r42] Staneck J. L. Roberts G. D. **(**1974**).** Simplified approach to identification of aerobic actinomycetes by thin-layer chromatography. Appl Microbiol 28, 226–231 4605116 10.1128/am.28.2.226-231.1974 PMC186691

[r43] Tamura K. Peterson D. Peterson N. Stecher G. Nei M. Kumar S. **(**2011**).** mega5: molecular evolutionary genetics analysis using maximum likelihood, evolutionary distance, and maximum parsimony methods. Mol Biol Evol 28, 2731–2739 10.1093/molbev/msr121 21546353 PMC3203626

[r44] Tindall B. J. **(**1990a**).** A comparative study of the lipid composition of *Halobacterium saccharovorum* from various sources. Syst Appl Microbiol 13, 128–130 10.1016/S0723-2020(11)80158-X

[r45] Tindall B. J. **(**1990b**).** Lipid composition of *Halobacterium lacusprofundi**.* FEMS Microbiol Lett 66, 199–202 10.1111/j.1574-6968.1990.tb03996.x

[r46] Wayne L. G. Brenner D. J. Colwell R. R. Grimont P. A. D. Kandler O. Krichevsky M. I. Moore L. H. Moore W. E. C. Murray R. G. E. **& other authors (**1987**).** International Committee on Systematic Bacteriology. Report of the ad hoc committee on reconciliation of approaches to bacterial systematics. Int J Syst Bacteriol 37, 463–464 10.1099/00207713-37-4-463

[r47] Westerberg K. Elväng A. M. Stackebrandt E. Jansson J. K. **(**2000**).** *Arthrobacter chlorophenolicus* sp. nov., a new species capable of degrading high concentrations of 4-chlorophenol. Int J Syst Evol Microbiol 50, 2083–2092 10.1099/00207713-50-6-2083 11155983

[r48] Whiton R. S. Lau P. Morgan S. L. Gilbart J. Fox A. **(**1985**).** Modifications in the alditol acetate method for analysis of muramic acid and other neutral and amino sugars by capillary gas chromatography-mass spectrometry with selected ion monitoring. J Chromatogr A 347, 109–120 10.1016/S0021-9673(01)95474-3 4086626

[r49] Yancey P. H. Clark M. E. Hand S. C. Bowlus R. D. Somero G. N. **(**1982**).** Living with water stress: evolution of osmolyte systems. Science 217, 1214–1222 10.1126/science.7112124 7112124

[r50] Yassin A. F. Spröer C. Siering C. Hupfer H. Schumann P. **(**2011**).** *Arthrobacter equi* sp. nov., isolated from veterinary clinical material. Int J Syst Evol Microbiol 61, 2089–2094 10.1099/ijs.0.026690-0 20870884

